# Dynamic Volumetric Imaging of Mouse Cerebral Blood Vessels In Vivo with an Ultralong Anti-Diffracting Beam

**DOI:** 10.3390/molecules28134936

**Published:** 2023-06-23

**Authors:** Yong Guo, Luwei Wang, Ziyi Luo, Yinru Zhu, Xinwei Gao, Xiaoyu Weng, Yiping Wang, Wei Yan, Junle Qu

**Affiliations:** State Key Laboratory of Radio Frequency Heterogeneous Integration (Shenzhen University), College of Physics and Optoelectronic Engineering, Key Laboratory of Optoelectronic Devices and Systems of Ministry of Education and Guangdong Province, Shenzhen University, Shenzhen 518060, China; 1800284004@email.szu.edu.cn (Y.G.); wanglowell@szu.edu.cn (L.W.); luoziyi2020@email.szu.edu.cn (Z.L.); 2150453014@email.szu.edu.cn (Y.Z.); 2250453008@email.szu.edu.cn (X.G.); xiaoyu@szu.edu.cn (X.W.); ypwang@szu.edu.cn (Y.W.); jlqu@szu.edu.cn (J.Q.)

**Keywords:** volumetric imaging, ultralong anti-diffracting, SBR, mouse brain blood vessels, blood flow velocity

## Abstract

Volumetric imaging of a mouse brain in vivo with one-photon and two-photon ultralong anti-diffracting (UAD) beam illumination was performed. The three-dimensional (3D) structure of blood vessels in the mouse brain were mapped to a two-dimensional (2D) image. The speed of volumetric imaging was significantly improved due to the long focal length of the UAD beam. Comparing one-photon and two-photon UAD beam volumetric imaging, we found that the imaging depth of two-photon volumetric imaging (80 μm) is better than that of one-photon volumetric imaging (60 μm), and the signal-to-background ratio (SBR) of two-photon volumetric imaging is two times that of one-photon volumetric imaging. Therefore, we used two-photon UAD volumetric imaging to perform dynamic volumetric imaging of mouse brain blood vessels in vivo, and obtained the blood flow velocity.

## 1. Introduction

A functional vascular supply plays a critical role in the efficient transportation of nutrients, fluids, gases, signaling molecules, and circulating cells between tissues and organs within mammalian systems [1,2,3,4,5]. Therefore, comprehending vascular development and function is crucial for comprehending both normal and diseased physiology. Dynamic volumetric imaging of cerebral vessels in vivo can reveal the structural dynamics and functional changes of samples in real time [6,7,8,9]. In conventional laser scanning fluorescence microscopy, the widely used modality for volumetric imaging is to serially capture optical section images by changing the observation plane, then reconstructing these images into a 3D image. Each optical section image displays focus information from different planes of the sample. The optical sectioning function is realized by confocal detection of fluorescent specimens using pinholes [10,11,12] or multiphoton excitation processes [13,14,15,16]. However, 3D image acquisition based on point scanning requires changing the position of the sample relative to the objective lens (moving the sample or moving the objective), which requires a certain response time, limits the acquisition speed, and prevents real-time observation of multiple dynamic events at different depths. Therefore, improving the acquisition speed of 3D volumetric images is of great significance.

To achieve this goal, various optical 3D imaging techniques based on different principles have been developed to greatly improve the performance of optical microscopes. Imaging methods such as multifocal scanning [17,18,19], line scanning [20,21,22,23], and light sheet illumination [24,25,26,27] can directly improve imaging speed. For example, optical slice images are captured with a frame rate of 1 kHz by rotating a disk [17], or of over 10 kHz by line scanning [20]. However, the scanning focusing method involves acquiring a z-stack of images in chronological order, which still requires the construction of 3D volumetric images, which becomes a bottleneck for real-time 3D imaging. Compared with the point-scanning imaging mode, the imaging speed of the light sheet microscope is much faster than that of the laser scanning confocal microscope, which makes it possible to study some high-speed and fine life processes. Unfortunately, the unique orthogonal optical path design imposes harsh conditions on the sample, hindering large deep tissue or living animal observation.

Recently, other techniques to extend the depth of focus (DOF) provide a new idea for fast 3D imaging, such as using anti-diffracting Bessel and Airy beams, which have been explored to increase the acquisition speed by converting the axial long depth of focus information into a two-dimensional (2D) image [28,29,30,31,32,33,34]. The extended focal length vastly extends the depth of field, providing a fast volumetric acquisition speed and avoiding a large amount of time wasted by axial scanning. Therefore, these techniques are widely recognized as a rapid volumetric acquisition method [35,36,37,38,39,40,41,42,43] that is commonly used for volumetric imaging of thick fixed tissue samples [32,35,36,39] and functional imaging of neural activity in the living brain [6,28,44]. Nevertheless, the volumetric approach rapidly increases the acquisition speed by converting the large axial DOF information into a 2D image. The depth information will be lost in the recorded images, and is only suitable for sparsely labeled samples (For example, the vertical projection of the Bessel beam, with a needle-like intensity distribution, results in the inability to provide information along the z-axis). For volumetric imaging of the cerebral vasculature with a crisscross distribution, this approach will no longer be effective because too much axial information is projected together. Although the depth information can be recovered by additional image acquisition (z-stack of the same field of view [31,36,39,45] or image post-processing [29,32,41,44,45]), the image quality is inevitably severely affected by sidelobes, and the finite focal length results in depth limitations in volumetric imaging (as far as we know, the maximum depth of single frame volumetric imaging in vivo is only 60 μm [6,28]), which hinders deep dynamic observation and hemodynamic analysis. To overcome the limited axial DOF, our group has developed an ultralong anti-diffracting (UAD) beam with adjustable axial focal length [46] and reported a volumetric one-photon excitation microscope using UAD beams (OPM-UAD) for illumination [47]. Volumetric imaging at a thickness of 120 μm was achieved within a transparent zebrafish. Similarly, the volume image was also affected by the sidelobe of UAD, resulting in a decrease in the signal-to-back ratio (SBR) of volume images. The poor SBRs of volumetric images and limited capture depth of volumetric imaging are not favorable for real-time observation and hemodynamics analysis.

In this work, we developed a two-photon excitation microscope using UAD beams for illumination (TPM-UAD). The performance of the TPM-UAD and the OPM-UAD was evaluated by volumetric imaging of mouse cerebral vessels in vivo. The results show that the TPM-UAD can capture deeper information and improve the volumetric image SBR by twofold compared with the OPM-UAD. Furthermore, the TPM-UAD enables us to quickly locate vessels of interest and obtain the blood flow velocity.

## 2. Experimental Setup

**Figure 1:**
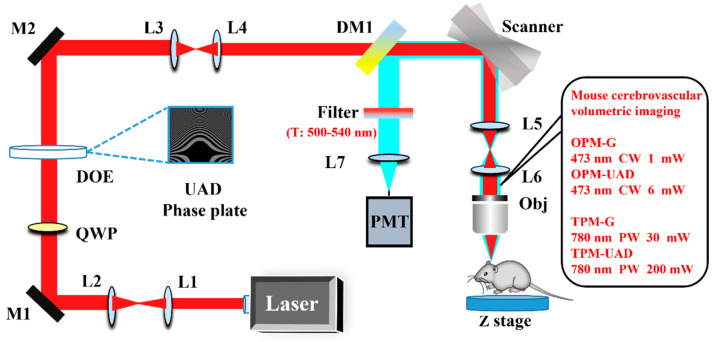
Schematic of the experimental setup (L1–L7: lenses; QWP: quarter-wave plate; DOE: diffractive optical element; DM: dichroic mirror; Scanner: X-Y linear scanner; M1–M2: mirrors; PMT: photomultiplier tube; Obj: objective; Z stage: motorized precision translation stage). CW: continuous wave; PW: pulsed wave.

The OPM-UAD and TPM-UAD imaging systems share a set of experimental setups, and a schematic of the experimental setups is shown in Figure 1. As shown in Figure 1, a tunable Chameleon laser (pulse laser with a 4 W power, an 80 MHz frequency, and a 140 fs pulse width) provides 780 nm femtosecond (CW: 473 nm for the OPM-UAD) Gaussian light, which is expanded by a pair of lenses (L1: 100 mm, L2: 300 mm). Then, the collimated linearly polarized Gaussian beam passes through a quarter-wave plate (QWP) (half-wave plate for the OPM-UAD, to adjust the polarization state of linearly polarized light to match the spatial light modulator (SLM)) and becomes a circularly polarized beam. The circularly polarized (linearly polarized) Gaussian beam is modulated by a custom diffractive optical element (DOE) (SLM for the OPM-UAD) integrated into the UAD phase plate, which converts the Gaussian beam into the UAD beam. The DOE is conjugated to a pair of X-Y galvanometric scanning mirrors (Cambridge Technology, 6210H) through a 4f lens system (L3:200 mm, L4:100 mm). X-Y galvanometric scanning mirrors are employed to scan the sample with the UAD beam, which provides a 1 Hz frame (512 × 512 pixels) rate. The scanning mirrors are further conjugated to the rear aperture of a high-numerical-aperture water-immersion objective (Leica, 1.0 NA, 20×) via a second 4f lens system (L5, 75 mm, L6, 200 mm). The fluorescence signal is collected by the same objective, and separated from the excitation optical path by a dichroic mirror (Chroma, ZT750RDC for the TPM-UAD, T505LPXR for the OPM-UAD). Then, the fluorescence signal is focused by a lens (L7, 200 mm) onto a photomultiplier tube (PMT, Hamamatsu, H7422-40). A bandpass filter (Chroma ET520/40) is introduced before the PMT to remove any residual excitation light. The DOE is mounted on a collapsible base, so the setup can be switched between Gaussian and UAD modes. The Gaussian and UAD modes can be switched by closing the SLM or loading the UAD mask onto the SLM in the OPM-UAD.

## 3. Results and Discussion

**Figure 2:**
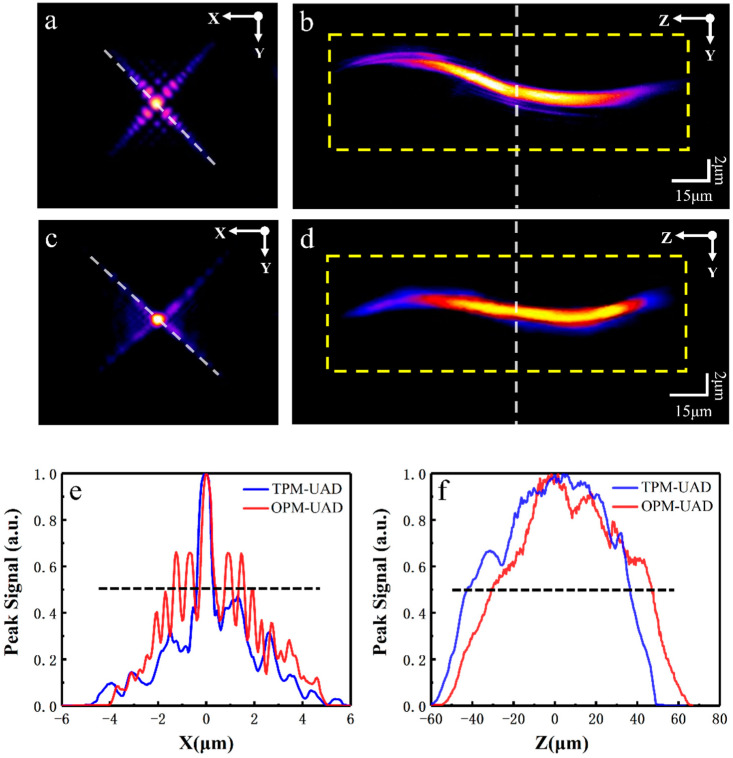
Images of a fluorescent bead were obtained in the OPM-UAD and TPM-UAD modes with a NA = 1.0 objective. (**a**,**c**) are lateral images obtained in the two modes; (**b**,**d**) are axial images obtained in the two modes. (**e**) Intensity profiles in the lateral images along the white dotted lines in (**a**,**c**). (**f**) Maximum signal intensity along the axial direction for (**b**,**d**). Red and blue lines represent the OPM-UAD beams and the TPM-UAD beams, respectively. Both signal intensities of the two modes are normalized.

In the whole experiment, we first investigated the spot imaging properties under the OPM-UAD and TPM-UAD imaging modes using fluorescent beads (excitation: 505 nm; emission: 515 nm) with a diameter of 0.2 μm. Figure 2a,c show lateral images of the fluorescent beads under the OPM-UAD and TPM-UAD modes, respectively. Figure 2e shows the intensity profiles along the white dotted lines in Figure 2a,c. As shown in Figure 2e, the full-width at half-maximum (FWHM) values of the spot (main lobe) images for the OPM-UAD and TPM-UAD mode beams are 0.4 μm and 0.5 μm, respectively. Although the spot diameter of the TPM-UAD is slightly larger than that of the OPM-UAD, the difference is not large. However, the ratio of the OPM-UAD beam first-sidelobe fluorescence intensity to the main-lobe fluorescence intensity is higher than that of the TPM-UAD beam (68% for the OPM-UAD; 40% for the TPM-UAD), and the intensity ratio of the second sidelobe is also larger (65% for the OPM-UAD; 30% for the TPM-UAD). In other words, the OPM-UAD sidelobes are more visible than the TPM-UAD sidelobes, which means that the OPM-UAD volumetric image is more blurred. Furthermore, we obtained the axial light field distribution information using z-axis scanning to obtain more information on the spot images in the two modes. Figure 2b,d show the axial light field distributions under the OPM-UAD and TPM-UAD modes, respectively, and the maximum signal intensity along the axial direction, which is taken as the selected longitudinal average strength value in the yellow dashed box, is shown in Figure 2f. The axial FWHMs are 85 ± 1 μm and 82 ± 1 μm for the OPM-UAD and TPM-UAD modes, respectively. Thus, the axial FWHMs of the two modes are not very different. In the axial light field distribution, we can see the OPM-UAD sidelobes (Figure 2b), while the TPM-UAD sidelobes can hardly be seen (Figure 2d). Due to the focal length of the UAD beam having a symmetrically curved trajectory, the projection directions of the volumetric image and UAD sidelobes are not perpendicular, and the volumetric image will be slightly blurred; thus, the volumetric image SBR will be different between the two modes.

**Figure 3:**
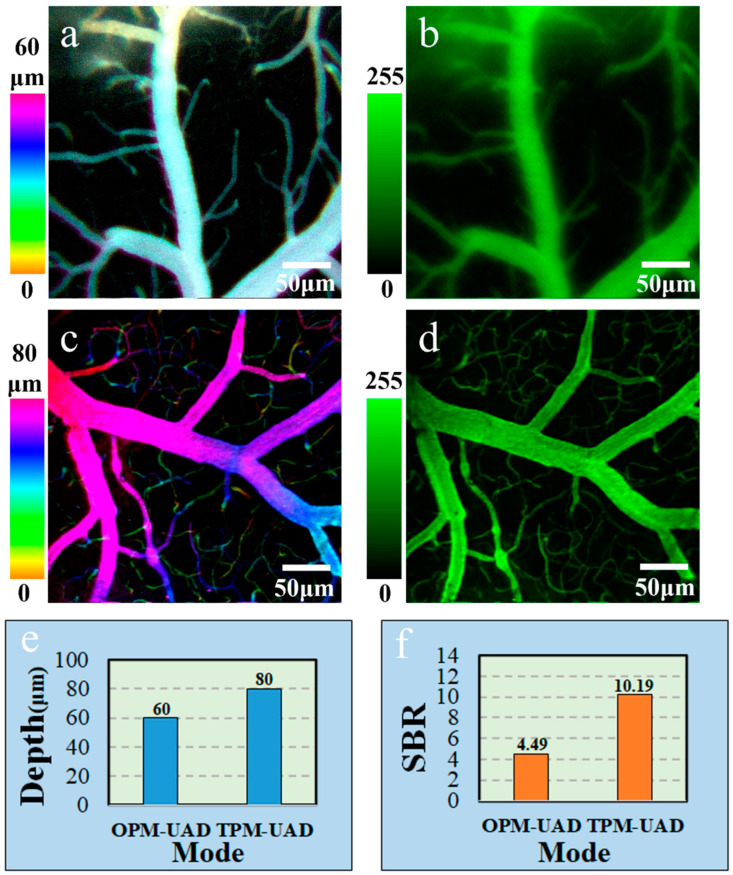
Mouse cerebrovascular volumetric imaging. (**a**) Projection of the Gaussian image stack of mouse cerebral vessels obtained in the OPM-G mode, color-coded by depth. Step size: 1 μm. (**b**) Single-frame UAD volumetric image of mouse cerebral vessels obtained in the OPM-UAD mode. (**c**) Projection of the Gaussian image stack of mouse cerebral vessels obtained in the TPM-G mode, color-coded by depth. Step size: 1 μm. (**d**) Single-frame UAD volumetric image of mouse cerebral vessels obtained in the TPM-UAD mode. (**e**) Depth comparison of single-frame volumetric images obtained in different modes. (**f**) SBR comparison of single-frame volumetric images obtained in different modes. Laser power: 1 mW for OPM-G mode, 6 mW for OPM-UAD mode, 30 mW for TPM-G mode and 200 mW for TPM-UAD mode.

Next, we performed imaging of mouse cerebral vasculature in four modes: OPM-G (one-photon Gaussian beam), TPM-G (two-photon Gaussian beam), OPM-UAD, and TPM-UAD modes. Figure 3a,c show the projection of the OPM-G image stack (60 frames with a 1 μm step size) and TPM-G image stack (80 frames with a 1 μm step size) of the mouse cerebral vessels labeled with FITC-dextran, respectively. Such 3D image stacks usually take around 1 min and 1.5 min to record, respectively. The effective imaging depths of the two modes are approximately 60 μm and 80 μm, respectively, coded in different colors to show the 3D projection of in vivo mouse brain vessels. Figure 3b,d show OPM-UAD and TPM-UAD volumetric images of in vivo mouse cerebral vessels. Such a frame volume image takes only 1 s to record. From Figure 3b,d, we can see that the information of the volumetric images almost covers that in Figure 3a,c, respectively. Therefore, a single 2D volumetric image in the OPM-UAD and TPM-UAD modes could be used to replace the projection image of a 3D image stack with 60 frames in OPM-G images and 80 frames in TPM-G images, which would greatly improve the volumetric imaging acquisition speed and effectively avoid motion blur in vivo imaging. For single-frame volumetric images, the TPM-UAD improves the imaging depth compared to the OPM-UAD. The imaging depth is 60 μm for the OPM-UAD mode and 80 μm for the TPM-UAD mode, showing a 1.3-fold improvement in the imaging depth of the single-frame volumetric image for the TPM-UAD (Figure 3e). In addition, the TPM-UAD can improve the SBR of the volumetric image of mouse cerebral vascular structures (Figure 3d). The SBRs, calculated as the ratio of the average intensity value of pixels in the signal area (in the image) to that of pixels in the background area, are 4.49 ± 1 for the OPM-UAD and 10.19 ± 2 for the TPM-UAD, showing a twofold improvement in background suppression for the TPM-UAD (Figure 3f).

**Figure 4:**
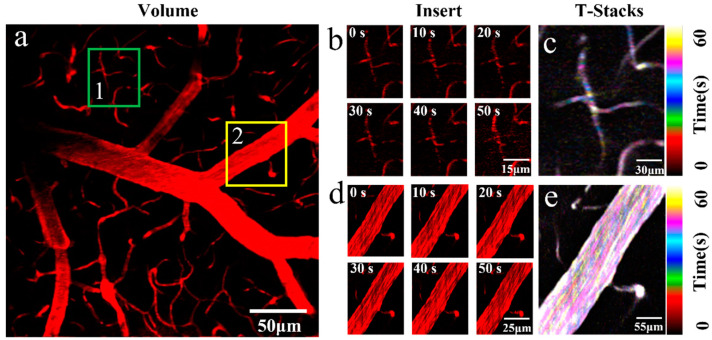
Dynamic volume monitoring. (**a**) Mouse cerebrovascular volumetric imaging in the OPM-UAD mode. (**b**) Dynamic process of small vessels (0 s to 50 s). (**c**) Stacked images of small vessels at different times (T-Stacks), color-coded by time. (**d**) Dynamic process of big vessels (0 s to 50 s). (**e**) Stacked images of big vessels at different times, color-coded by time.

Volumetric imaging demonstrated the feasibility of TPM-UAD in vivo imaging, and dynamic processes are an important part of volumetric imaging research. Therefore, we performed dynamic volume monitoring of blood vessels in the TPM-UAD mode in Figure 4 (see Video S1 for more details). As shown in Figure 4a, the different sizes of cerebral vessels of the mouse were captured in TPM-UAD mode. In order to study the mouse cerebral vascular structures information in the volume image in detail, two vessels of different sizes were selected as mouse cerebral vascular structures for study (the green boxed areas are small vessels and the yellow boxed areas are big vessels). In Figure 4b, it can be seen that the fluorescence intensity of blood vessels changes at different times (0 s–50 s), which proves that the UAD beam is feasible to study the dynamic process of blood flow in small vessels. However, the intensity map has little change, so the dynamic change process of blood flow over time cannot be well studied. Therefore, the images with different blood flow times were endowed with different pseudo-colors and stacked (Figure 4c). When the color of the overlapping images is white, it indicates that there is no obvious dynamic process in this area, and when the color of the overlapping images is another color, it indicates the blood flow movement process. Therefore, it can be proved more intuitively that TPM-UAD can effectively study the blood flow motor process. Consistent with the study of the blood flow process in small vessels, TPM-UAD is also proved to be a powerful approach to study the dynamic process of blood flow in large vessels in Figure 4d,e. Above all, TPM-UAD can be used to study the blood flow process in vivo.

**Figure 5:**
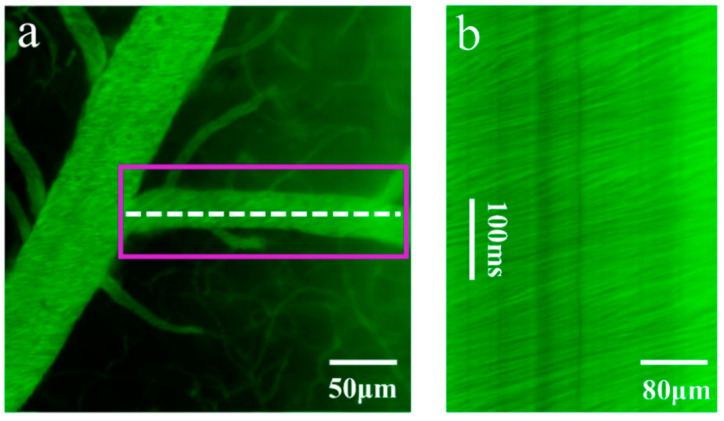
Blood flow speed measurements in blood vessels. (**a**) Mouse cerebrovascular volumetric imaging under the TPM-G mode. We recorded line scans along the central axis of the vessel at a rate of ~2 ms per line, and the line scan trajectory is marked by the white dashed line. (**b**) x (spatial dimension)-t (temporal dimension) dataset from the line scans.

Real-time visualization of the cerebral vasculature and hemodynamics is the key to understanding brain physiology and pathology [48,49,50,51,52,53,54]. However, there is a need to find suitable target vessels (a certain radial direction) for vascular velocity measurements based on linear scanning methods, and conventional imaging methods cannot achieve radial direction real-time visualization [55,56,57,58,59,60,61]. One major advantage of in vivo TPM-UAD imaging is the capacity to image physiological dynamics, providing large depth information and high-SBR volumetric images in real time. Hence, we performed blood flow speed measurements (Figure 5). First, under the TPM-UAD mode, volumetric imaging was carried out. Benefiting from the ultralong field depth of the UAD, we could see all the information in the axial depth of focus in real time (Figure 5b), so we could quickly locate a blood vessel suitable for measurement (fuchsia box). Then, the TPM-G mode was switched to, and standard line scanning technology was used to measure the blood flow speed. The data recorded by the line scan are shown in Figure 4c. Line-scanning particle image velocimetry (LS-PIV) was used to calculate the blood flow velocity [62] (see Supplementary Materials). The speed of the blood flow to the large diameter vessels in the cortex was measured to be 1.48 mm/s, with a standard deviation of 0.5 mm/s.

For in vivo imaging, mice (C57, 8 months old, 20 g) were anesthetized using intraperitoneally injected urethane (150 mg/100 g animal weight), and a craniotomy was performed above the parietal cortex. The dura was left intact. A glass coverslip was affixed to the skull using dental cement to seal the craniotomy. Two hundred microliters of a dye mixture (7 kDa FITC-dextran, each dissolved at 2.5% *w*/*v* in saline) were retro-orbitally injected to label the vasculature. All animal procedures were reviewed and approved by the Experimental Animal Ethics Committee of Shenzhen University.

In order to ensure the comparability of the experimental conditions, the excitation laser power under different modes was controlled in our experiment. In mouse cerebral vascular volumetric imaging, the excitation laser power of OPM-G, TPM-G, OPG-UAD and TMG-UAD were 1 mW, 30 mW, 6 mW and 200 mW, respectively. The excitation laser power of two-photon is apparently higher than that of one-photon, the main reason is that the absorption cross-section of two-photon excitation is several orders of magnitude smaller than that of one-photon excitation, so two-photon excitation requires higher excitation power [63]. In addition, the axial FWHM of the UAD beam is longer than that of the Gaussian beam (82 μm for the TPM-UAD; 4 μm for the TPM-G). Therefore, increasing the laser power in the TPM-UAD mode is necessity. In order to acquire a volumetric image of considerable depth, we increased the excitation laser power to 200 mW in TPM-UAD mode.

## 4. Conclusions

In this work, we demonstrated volumetric imaging in the OPM-UAD and TPM-UAD modes of the brain of a mouse in vivo. In the UAD mode (OPM-UAD and TPM-UAD), the elongated focal length of the UAD beam provides a large depth of field, which increases the volumetric imaging rate. Meanwhile, unlike the traditional Gaussian mode (OPM-G and TPM-G), which is very sensitive to the position of the sample, the OPM-UAD and TPM-UAD are more robust against the axial motion of the samples, since they capture all axial images within the effective focal length and avoid additional noise as well as time waste by skipping axial scanning. However, the poor imaging performance of the OPM-UAD is due to the use of short-wavelength excitation light and the presence of UAD sidelobes. Fortunately, the TPM-UAD volumetric imaging performance is significantly improved, by 1.3-fold in the volumetric imaging depth and twofold in the image SBR. Two possible factors combine to contribute to the results of this paper. On the one hand, the use of a longer wavelength (780 nm) reduces the scattering of excitation light by tissue, making it easier for the excitation light to penetrate deep tissue and reach the interior of the tissue; on the other hand, the two-photon nonlinear excitation suppresses the generation of sidelobe signals. In addition, the real-time capture of large depth information provides great convenience for quickly finding target blood vessels in blood flow velocimetry. In conclusion, the TPM-UAD is a potential tool for real-time monitoring of deep biological activities in a large volume.

## Data Availability

The raw data supporting the conclusions of this article will be made available by the authors, without undue reservation.

## Supplementary Materials

The following supporting information can be downloaded at: https://www.mdpi.com/article/10.3390/molecules28134936/s1, Video S1. Dynamic two-photon volumetric imaging of mouse cerebral blood vessels with UAD beams.
