# Supplementary material for: Dynamic Volumetric Imaging of Mouse Cerebral Blood Vessels In Vivo with an Ultralong Anti-Diffracting Beam
Source: Molecules. 2023 Jun 23;28(13):4936. doi: 10.3390/molecules28134936 (PMC10343797; doi:10.3390/molecules28134936)
Supplement: Supplementary file 1 [file molecules-28-04936-s001.zip › molecules-2435678-supplementary.pdf]

Supplementary Information

Dynamic Volumetric Imaging of Mouse Cerebral Blood Vessels In Vivo with an Ultralong Anti-Diffracting Beam

Yong Guo, Luwei Wang, Ziyi Luo, Yinru Zhu, Xinwei Gao, Xiaoyu Weng, Yiping Wang, Wei Yan * and Junle Qu

State Key Laboratory of Radio Frequency Heterogeneous Integration (Shenzhen University), College of Physics and Optoelectronic Engineering, Key Laboratory of Optoelectronic Devices and Systems of Ministry of Education and Guangdong Province, Shenzhen University, Shenzhen 518060, China; 1800284004@email.szu.edu.cn (Y.G.); wanglowell@szu.edu.cn (L.W.); luoziyi2020@email.szu.edu.cn (Z.L.); 2150453014@email.szu.edu.cn (Y.Z.); 2250453008@email.szu.edu.cn (X.G.); xiaoyu@szu.edu.cn (X.W.); ypwang@szu.edu.cn (Y.W.); jlqu@szu.edu.cn (J.Q.)

* Correspondence weiyan@szu.edu.cn

Methods for Measuring Blood Flow

The brain is one of the most important organs of the human body. The blood flow velocity in the brain is an important parameter of blood supply and hemodynamics in the brain. The measurement of this parameter can quantitatively reflect the physiological and pathological conditions of the brain, such as nerve cell activity, cerebrovascular embolism, and peripheral blood flow abnormalities caused by Senile Dementia [1–5]. The ability to measure blood velocities is critical for studying vascular development, physiology, and pathology. Therefore, how to quantitatively measure brain blood flow velocity at the single vessel level has been a research hotspot in brain science. Techniques based on different principles have been developed for hemodynamic analysis. At the macroscopic scale, intrinsic optical imaging [6] and laser speckle [7] imaging can obtain information about blood oxygen and blood flow changes, but they are not suitable for blood flow measurement of blood vessels below the cortical surface. Laser Doppler imaging, functional magnetic resonance imaging, optical coherence tomography, and functional ultrasound can penetrate beyond the surface of the cortex to image deep inside [8–12], but all have the low spatial resolution. Confocal laser scanning microscopy (CLSM) has been widely used at the microscopic scale for vascular blood flow velocity measurement. The use of line scanning and single-photon excitation imaging, combined with automatic detection and fluorescent-labeled red blood cell (RBC) tracking algorithms, has increased the feasibility of blood flow analysis at the single-vessel level. However, the depth of single-photon imaging is limited, and blood flow velocity can only be measured at the surface of blood vessels. Two-photon laser scanning microscopy (TPLSM) has shown tremendous promise in analyzing blood velocities hundreds of micrometers deep in animals with cellular resolution. For this reason, TPLSM has been the tool of choice for studying hemodynamics at high resolution in mice [13–15].

In a mouse model, TPLSM can image fluorescent labeling deep in the brain with spatial resolution better than 1 μm. For in vivo studies, fluorescent labels can be endogenous or exogenous dyes, but many studies take advantage of the specificity of labeling specific cells or structures using genetic strategies. For the brain, the easiest way to label the blood vessels is an intravenous injection (usually in rodents via the tail or retro-orbitally) of dextran-conjugated dyes [14,16]. Circulating cells, including red and white blood cells, do not take up the dextran-conjugated dye, so they appear as dark patches within the vessels. TPLSM is a scanning microscope that moves the focus of a laser through the sample and measures the fluorescence produced at each spot. However, most TPLSM scan across the image region one line at a time and require at least 0.5 ms per line. The majority of red blood cells in capillaries are moving fast enough that full frame images will not capture the same blood cells in successive frames, thus making it impossible to quantify blood flow speed. To increase time resolution the laser focus is repeatedly scanned along the length of a vessel segment, called a "line-scan," instead of scanning a full image plane, essentially capturing a very thin image in the middle of the blood vessel (Figure S1). Scan speeds are generally fast enough that they will easily capture the same red blood cells in multiple successive line scans, thus enabling the speed of the cells to be measured. This data is often displayed by aligning the sequential line scans so that time is along the vertical axis and the path is along the vessel on the

horizontal axis, also known as a kymograph, or space-time image [17]. A stationary object, which is always in the same part of the vessel, will result in a straight, vertical line. Red blood cells moving along the length of the vessel generate diagonal streaks. The slower the cell moves, the more vertical the streak. The faster the cell moves, the more horizontal the streak. The speed of the red blood cell along the vessel is proportional to the inverse of the slope of the streak, or the cotangent of the angle made with the horizontal axis. We can measure the speed by measuring the angle of these streaks (Figure S1).

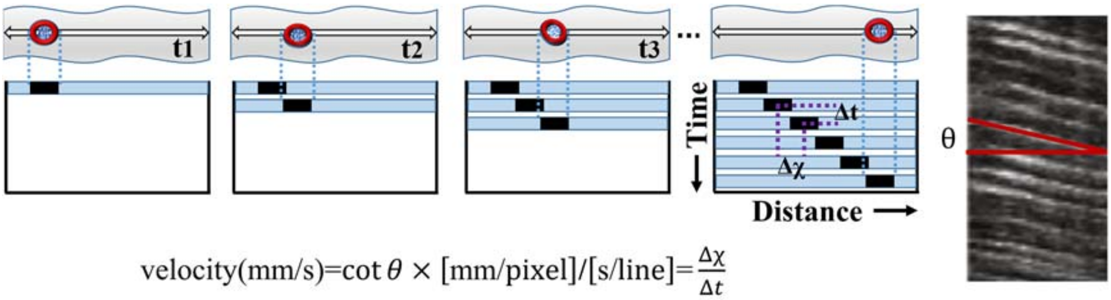

Figure. S1. Schematic of the axial linear scan

Quantifying blood flow velocity by determining the Angle of the fringes is suitable for calculating slow blood flow velocity. In measurements of arteries or large diameter vessels with fast blood flow rates, red blood cells move too quickly to be tracked individually as continuous streaks in the space-time data. A line-scanning particle image velocimetry (LS-PIV) was reported in reference [18] to calculate the velocity. the LS-PIV determines the RBC displacements between pairs of line-scans using spatial cross-correlation analysis. This approach enables the quantification of blood velocities from capillaries to high-flow abnormalities deep within the living animal. Quantitative peak blood flow velocity up to 84 mm/s.

The Principle Of LS-PIV

RBCs appear dark amid fluorescently labeled plasma. Two successive line scans are recorded along the central axis of the vessel, capturing the displacement ($\Delta\chi$) of a red blood cell across time interval Δt. The line scans are Fourier-transformed, cross-correlated with a symmetric phase-weighted filtering operation, and inverse Fourier-transformed. A peak detection operation is performed to locate the correlation peak corresponding to the red blood cell displacement, which is converted to velocity by dividing by the time interval between the scans ($V(t)= \Delta\chi/\Delta t$). The specific steps of the LS-PIV algorithm are shown in Figure S2.

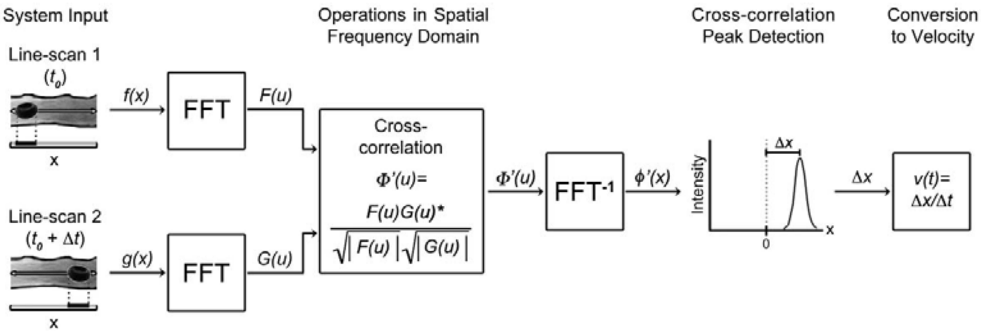

Figure. S2. Illustration of generalized LS-PIV[18].

Supplementary References

1. Ivanov, K. P.; Kalinina, M. K.; Levkovich, Y. I.; Blood flow velocity in capillaries of brain and muscles and its physiological significance. *Microvasc. Res.* **1981**, *22*,143–155.
2. Emmanuelle, C.; Pascale, T.; Jérôme, L.; Mathieu, D.; Thomas, K.; Serge, C.; The relationship between blood flow and neuronal activity in the rodent olfactory bulb. *J. Neurosci*. **2007**, *27*, 6452–6460.
3. Ungersböck, K.; Heimann, A.; Kempski, O.; Cerebral blood flow alterations in a rat model of cerebral sinus thrombosis. *Stroke* **1993**, *24*, 563–569.
4. Hays, C. C.; Zvinka, Z. Z.; Christina, E. W.; The utility of cerebral blood flow as a biomarker of preclinical Alzheimer's disease. *Cell. Mol. Neurobiol*. **2016,** *36*, 167–179.
5. Takashi, T.; Rong, Zh.; Cerebral blood flow in normal aging adults: cardiovascular determinants, clinical implications, and aerobic fitness. *J. Neurochem*. **2018,** *144*, 595–608.
6. Grinvald, A.; Lieke, E.; Frostig, R. D.; Gibert, C. D.; Wiesel, T. N.; Functional architecture of cortex revealed by optical imaging of intrinsic signals, *Nature* **1986**, *324*, 361–364.
7. Andrew, K. D.; Hayrunnisa, B.; Michael A. M.; David A. B.; Dynamic imaging of cerebral blood flow using laser speckle. *J. Cerebr. Blood F. Met*. **2001**, *21*, 195–201.
8. Beau, M. A.; Joel, G. H.; John, A. D.; Laser doppler imaging of activation-flow coupling in the rat somatosensory cortex. Neuroimage **1999**, *10*, 716–723.
9. Ogawa, S.; Tank, D. W.; Menon, R.; Ellermann, J. M.; Kim, S. G.; Merkle, H.; Ugurbil, K.; Intrinsic signal changes accompanying sensory stimulation: functional brain mapping with magnetic resonance imaging. *PNAS* **1992**, *89*, 5951–5955.
10. Srinivasan, V. J.; Sakadzic, S.; Gorczynska, I.; Yaseen, A.; Fujimoto, J.; Boas, D.; Depth-resolved microscopy of cortical hemodynamics with optical coherence tomography. *Opt. Lett*. **2009**, *34*, 3086–3088.
11. Hu, S.; Wang. L. H.; Photoacoustic imaging and characterization of the microvasculature. *J. Biomed. Opt*. **2010,** 15, 011101–011115.
12. Emilie, M.; Montaldo, G.; Cohen, I.; Baulac, M.; Fink, M.; Mickael, T.; Functional ultrasound imaging of the brain. *Nat. Methods* **2011**, *8*, 662–664.
13. David, K.; Mitra, P. P.; Helmchen, F.; Denk, W.; Fluctuations and stimulus-induced changes in blood flow observed in individual capillaries in layers 2 through 4 of rat neocortex. *PNAS* **1998**, *95*, 15741–15746.
14. Andy, Y. S.; Driscol, J. D.; Drew, P. J.; Nishimura, N.; Schaffer, C. B.; Kleinfeld, D.; Two-photon microscopy as a tool to study blood flow and neurovascular coupling in the rodent brain; *J. Cerebr. Blood F. Met*. **2012**, *32*, 1277–1309.
15. Holtmaat, A.; Bonhoeffer, T.; Chow, D. K. Chuckowree, J.; Paola, V. D.; Hofer, S. B.; Hübener, M.; Keck, T.; Knott, G.; Wei-Chung A Lee, Mostany, R.; Mrsic-Flogel, T. D.; Nedivi, E.; Portera-Cailliau, C.; Svoboda, K.; Trachtenberg, J. T.; Wilbrecht, L.; Long-term, high-resolution imaging in the mouse neocortex through a chronic cranial window. *Nat. Protoc*. **2009**, *4*, 1128–1144.
16. Kobat, D.; Durst, M. E., Nishimura, N.; Wong, A. W.; Xu, C.; Deep tissue multiphoton microscopy using longer wavelength excitation. *Opt. Express* **2009**,*17*, 13354–13364.
17. Nishimura, N.; "Experimental Methods for Measuring Blood Flow in Brain Capillaries," Primer on Cerebrovascular Diseases. *Academic Press*, **2017**,339–343, https://doi.org/10.1016/B978-0-12-803058-5.00071-0.
18. Kim, T.N.; Goodwill, P.W.; Chen, Y.N.; Conolly, S.M.; Schaffer, C.B.; Liepmann, D.; Wang, R.A. Line-scanning particle image velocimetry: An optical approach for quantifying a wide range of blood flow speeds in live animals. *PLoS ONE*, **2012**, 7, e38590.
